# Electroporated recombinant proteins as tools for in vivo functional complementation, imaging and chemical biology

**DOI:** 10.7554/eLife.48287

**Published:** 2019-07-16

**Authors:** Amal Alex, Valentina Piano, Soumitra Polley, Marchel Stuiver, Stephanie Voss, Giuseppe Ciossani, Katharina Overlack, Beate Voss, Sabine Wohlgemuth, Arsen Petrovic, Yaowen Wu, Philipp Selenko, Andrea Musacchio, Stefano Maffini

**Affiliations:** 1 Department of Mechanistic Cell BiologyMax Planck Institute of Molecular PhysiologyDortmundGermany; 2 In-Cell NMR LaboratoryLeibniz Institute of Molecular Pharmacology (FMP Berlin)BerlinGermany; 3 Chemical Genomics CentreMax Planck SocietyDortmundGermany; 4 Department of ChemistryUmeå UniversityUmeåSweden; 5 Department of Biological RegulationWeizmann Institute of ScienceRehovotIsrael; 6 Centre for Medical Biotechnology, Faculty of BiologyUniversity Duisburg-EssenEssenGermany; Virginia TechUnited States; Utrecht UniversityNetherlands

**Keywords:** electroporation, recombinant protein, protein delivery, kinetochore, protein modificatin, chemical biology, Human

## Abstract

Delivery of native or chemically modified recombinant proteins into mammalian cells shows promise for functional investigations and various technological applications, but concerns that sub-cellular localization and functional integrity of delivered proteins may be affected remain high. Here, we surveyed batch electroporation as a delivery tool for single polypeptides and multi-subunit protein assemblies of the kinetochore, a spatially confined and well-studied subcellular structure. After electroporation into human cells, recombinant fluorescent Ndc80 and Mis12 multi-subunit complexes exhibited native localization, physically interacted with endogenous binding partners, and functionally complemented depleted endogenous counterparts to promote mitotic checkpoint signaling and chromosome segregation. Farnesylation is required for kinetochore localization of the Dynein adaptor Spindly. In cells with chronically inhibited farnesyl transferase activity, in vitro farnesylation and electroporation of recombinant Spindly faithfully resulted in robust kinetochore localization. Our data show that electroporation is well-suited to deliver synthetic and chemically modified versions of functional proteins, and, therefore, constitutes a promising tool for applications in chemical and synthetic biology.

## Introduction

The traditional divide between in vivo, in cellulo and in vitro approaches in modern molecular cell biology has progressively been overcome in recent years largely due to new conceptual and technical advancements. For instance, dissection and biochemical reconstitution of complex cellular pathways in vitro demonstrated great predictive value, facilitating and directing functional characterizations aiming to understand the endogenous cellular counterpart (e.g. Faesen et al., 2017; Yeeles et al., 2015). Chemical and synthetic biology, on the other hand, have generated tools for studying and manipulating biological pathways in creative new ways, with innovations that include, amongst others, genetic code expansion to introduce unnatural aminoacids with new functionalities into a protein of interest (Davis and Chin, 2012), the development of fluorescent dyes with potential for single molecule sensitivity and spanning a greater spectral range compared to genetically encoded proteins (Huang et al., 2009), and the design of allelic mutants of crucial enzymes that are specifically inhibited by small molecules that will not target the wild type allele (Bishop et al., 2001).

Cells are surrounded by plasma membranes, which constitute essential protective barriers for cellular integrity but impede the targeted delivery of biological macromolecules such as proteins. In turn, the controlled intracellular transduction of proteins has remained highly challenging. Historically, introduction of protein variants into cells has been achieved by transient transfection of DNA plasmids encoding these entities. Recipient cells then typically express the desired proteins at levels that vary between cell lines and individual cells (Kim and Eberwine, 2010). Owing to the ease of transient transfection procedures, the method is widely used. Stable transfection of cultured mammalian cells offers more uniform protein expression but the generation of stable clones is labor-intensive and time-consuming (Kim and Eberwine, 2010).

A significant fraction of cellular proteins exist within stable or dynamic multi-subunit assemblies (Charbonnier et al., 2008; Vidal et al., 2011; Aloy et al., 2004). Functional characterization of protein complexes in mammalian cells remains laborious especially when multiple subunits need to be manipulated (e.g. mutations or the introduction of fluorescent probes) for functional complementation studies. In such cases, multiple subunits must be co-expressed to similar levels while endogenous counterparts ought to be depleted, which is technically challenging and exposes a potential limitation of direct DNA-based delivery methods. Because robust tools for recombinant expression of protein complexes in heterologous systems, including bacteria and insect cells, are available in structural biology (Perrakis et al., 2011), the prospect of directly delivering these specimens into cells is highly attractive. In addition, defined protein modifications may be introduced in a target protein before intracellular delivery, thus enabling the exploitation of a repertoire of chemical biology tools for in-cell investigations.

The tools available for protein delivery, however, remain insufficiently explored (Fu et al., 2014). Historically, microinjection first emerged as the method of choice for the introduction of recombinant proteins into mammalian cells (Komarova et al., 2007). However, it requires considerable handling skills and a dedicated setup that are not routinely available, and is a low-throughput approach that limits delivery to a small number of targeted cells (typically in the range of tens to hundreds). For higher throughput, batch delivery methods such as cell-penetrating peptide (CPP) tags (Inomata et al., 2009), pore-forming bacterial toxins (Teng et al., 2016; Ogino et al., 2009) or osmocytosis (D'Astolfo et al., 2015) are available, but present uncertainties with regard to protein uptake efficiencies, undefined routes of internalization and intracellular localization, as well as varying degrees of cytotoxicity.

Batch electroporation (EP) is an alternative method to directly deliver proteins into cultured mammalian cells (Lambert et al., 1990; Chakrabarti et al., 1989; Vienken et al., 1978; Shi et al., 2018; Furuhata et al., 2019). EP-mediated protein transduction has surged in recent years with applications ranging from, cellular structural biology and the delivery of isotopically labeled proteins for in-cell NMR measurements (Theillet et al., 2016) to the introduction of immunoglobulins for targeting and degradation of endogenous proteins (Clift et al., 2017), and the delivery of ribonucleotide particles (RNPs) for CRISPR-Cas9-mediated gene editing (Kim et al., 2014; Lin et al., 2014; Liang et al., 2015; Hashimoto and Takemoto, 2015).

**Figure 1.**
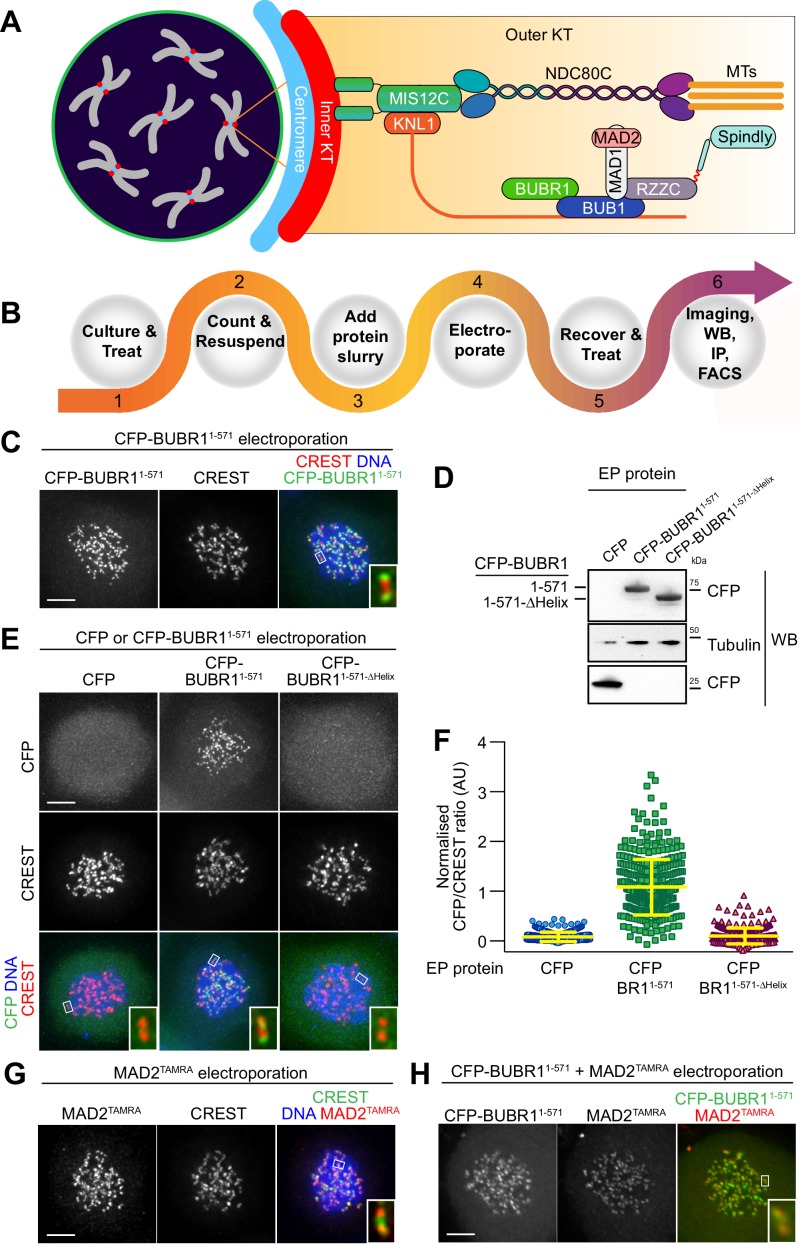
Delivery of spindle assembly checkpoint proteins by electroporation. (**A**) Schematic outline of a mitotic cell with condensed chromosomes (gray) and centromeres (blue). The inner kinetochore (red) recruits several outer kinetochore proteins (orange gradient box), including the MIS12 (MIS12C) and the microtubule (MT) binding complex NDC80 (NDC80C). In turn, these complexes recruit the protein KNL1, the spindle assembly checkpoint (SAC) components BUB1, BUBR1, MAD1 and MAD2, and the outer kinetochore proteins RZZ and Spindly. (**B**) Overview of the electroporation (EP) work-flow using adherent cells. (1) Cells are cultured under defined growth conditions according to experimental requirements. (2) Prior to EP, cells are harvested by trypsinization, washed in PBS, counted and resuspended in EP buffer. (3) Recombinant protein diluted in EP buffer is added to cell slurries at desired concentrations (this stage is called EP slurry). (4) Cells are pulsed between 1 and 3-times to allow for efficient delivery of recombinant proteins. (5) Cells are tripsinised and then washed twice to remove non-incorporated proteins, and then plated on cover slips or culture flasks to allow for recovery. (6) After a recovery period, cells are suitable for analysis with a number of different read-outs and applications, including, amongst others, imaging, western blotting, immunoprecititaion (IP) and FACS analysis. (**C**) Following EP of CFP-BUBR1^1-571^ and over-night recovery, cells were treated for 6 hr with nocodazole and then prepared for immunofluorescence analysis. Kinetochores were stained with the marker CREST and DNA with SiR-Hoechst-647. Insets represent magnifications of the boxed kinetochores. Scale bar = 5 µm. (**D**) CFP alone, CFP-BUBR1^1-571^ and the BUB1-binding-deficient mutant CFP-BUBR1^1-571-ΔHelix^ were electroporated under the same conditions used in C. Protein extracts generated from electroporated cells were subjected to western blotting analysis with the indicated antibodies. (**E**) CFP-BUBR1^1-571-ΔHelix^ fails to localize to kinetochores. Kinetochores were stained with the marker CREST and DNA with SiR-Hoechst-647. Scale bar = 5 μm. (**F**) Quantification of KT levels for electroporated CFP proteins from cells shown in E. Each symbol represents a single cell. Yellow lines indicate median values ± interquartile range. (**G**) SAC protein MAD2^TAMRA^ electroporated into cells display proper localization to unattached kinetochores. Kinetochores were stained with the marker CREST and DNA with DAPI. Scale bar = 5 μm. Insets represent magnifications of the indicated KT. (**H**) Simultaneous electroporation of CFP-BUBR1^1-571^ and MAD2^TAMRA^ results in proper localization to kinetochores of nocodazole-treated HeLa cells. Insets represent magnifications of the indicated kinetochore. Scale bar = 5 µm.

While these approaches seem promising, more experimental evidences regarding the structural and functional intactness of delivered proteins in cells and a comprehensive analysis of the robustness and reproducibility with which different mammalian cells can be targeted are needed. Here, we test EP as a delivery method for various proteins that work in the kinetochore, a multi-subunit assembly required for chromosome segregation (Musacchio and Desai, 2017). The kinetochore consists of at least 30 core subunits organized in various protein assemblies and structured in layers that emanate from a specialized centromeric chromatin interface, the inner kinetochore, and terminate at the outer kinetochore, where spindle microtubules are bound (Figure 1A). The primary function of kinetochores is to link chromosomes and spindle microtubules (MTs) (Pesenti et al., 2018). Kinetochores function also as signaling platforms for the spindle assembly checkpoint (SAC, also named mitotic or metaphase checkpoint), a feedback surveillance mechanism that monitors the correct state of kinetochore-microtubule (KT-MT) attachment to ensure timely and faithful chromosome segregation (Musacchio, 2015).

**Figure 1—figure supplement 1.**
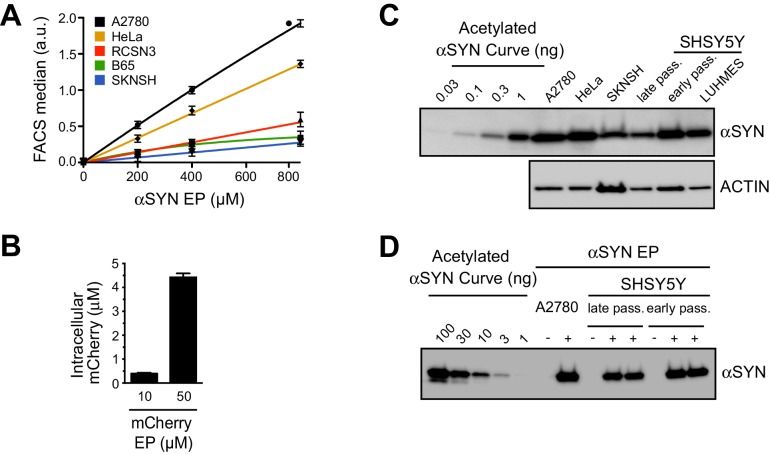
Measuring protein uptake levels upon electroporation of multiple cell lines. (**A**) Quantification of EP-mediated protein uptake by flow cytometry across different cell lines. Median fluorescence intensities (MFI) determined by flow cytometry of EP-processed cells harboring Atto488-labeled α-synuclein (αSYN) plotted against αSYN input concentrations in the respective EP mixtures. Linear uptake efficiencies are evident for all cell lines despite cell-type specific differences in general uptake properties. MFI values were corrected for average cell sizes and corresponding volumes. Lines correspond to linear fits connecting individual measurement points. Error bars are SDM from four independent samples. (**B**) Quantification of EP-mediated protein uptake by fluorimetric analysis. Extracts from HeLa cells electroporated with mCherry and analyzed with a fluorimeter show that uptake is proportional to the input protein concentration. Fluorescence intensity of protein extracts generated from a known number of cells that were electroporated with different amounts of recombinant mCherry were quantified with a fluorimeter and plotted against an input titration curve. The values obtained were subtracted for the intensity values measured from a mock EP sample. Graph represents mean values ± SD. (**C**) Western blotting analysis of whole cell lysates to detect endogenous αSYN levels in A2780, HeLa, SK-N-SH, early- and late-passage SH-SY5Y and undifferentiated LUHMES cells. Defined concentrations of recombinant N-terminally acetylated αSYN serve as input control. Average intracellular concentrations are in the range of 0.05 to 0.5 µM, calculated based on experimentally determined cell volumes (Theillet et al., 2016). (**D**) By comparison to the endogenous levels determined in panel C, cells electroporated with 400 µM αSYN in the EP slurry harbor ~100 fold higher levels of the recombinant protein (see input concentrations for reference).

**Figure 1—figure supplement 2.**
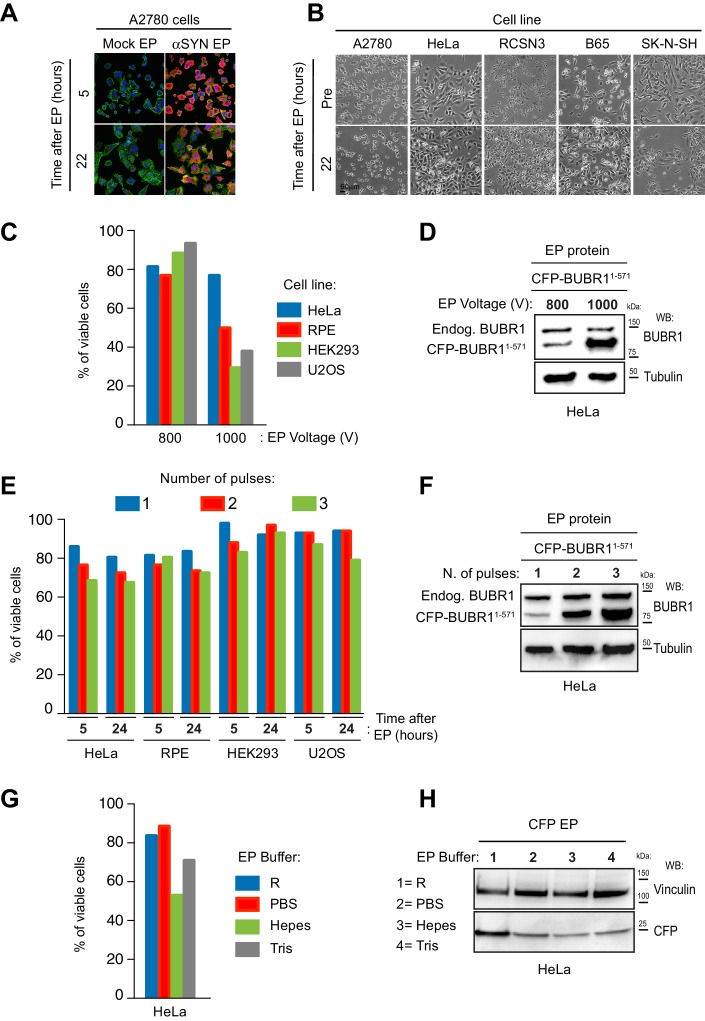
Analysis of cell viability and protein uptake in multiple cell lines electroporated with different EP settings. (**A**) Electroporation of αSYN in A2780 cells produces low levels of cell damage. Panels show anti-αSYN immunofluorescence detection in A2780 cells at different time-points following batch EP and recovery. DNA is shown in blue (DAPI), actin in green (phalloidin) and αSYN in red. Note that most cells harbor αSYN (high penetrance) and that intracellular protein levels are similar between cells (uniform delivery efficiency). 22 hr after EP, adherent cells regain their physiological cell morphologies, an indication that EP caused low cytotoxicity. Mock EP was carried out without recombinant αSYN in the EP mixture. (**B**) EP does not have major cytotoxic effects across different cell lines. Bright field analysis of neuronal (RCSN-3, B65, SK-N-SH) and non-neuronal (A2780, HeLa) cell lines electroporated with α-synuclein (αSYN) to inspect cell morphology and viability. (**C**) Viability of RPE, HEK293 and U2OS cells is sensitive to EP voltage. Cell lines were electroporated with recombinant CFP and then stained with Trypan blue to measure viability at 5 hr after EP. Settings used for the delivery were either 800V/2 × 25 msec pulses or 1000 V/2 × 35 msec pulses. (**D**) Western blotting analysis of whole cell lysates from HeLa cells electroporated with CFP-BUBR1^1-571^ and with voltage settings as in panel C. (**E**) Effect of the number of EP pulses on cell viability. Cells electroporated with recombinant CFP and using an increasing number of EP pulses were stained with Trypan blue to measure viability at the indicated time points after EP. HeLa cells were subjected to 1000 V/35 msec pulses while RPE, HEK293 and U2OS to 800 V/25 msec pulses. Values indicated for the two pulses/5 hr time points are the same shown in panel C. (**F**) Correlation between number of EP pulses and the cellular intake of recombinant protein. Western blotting analysis of HeLa cells electroporated with recombinant CFP-BUBR1^1-571^ using 1000 V and an increasing number of 35 msec pulses. Endog. = endogenous. (**G**) Effects of the EP buffers on cell viability. HeLa cells electroporated with CFP using the indicated buffers and settings of 1000 V/2 × 35 msec were analysed for viability with Trypan blues staining at 5 hr following EP. (**H**) Effect of different EP buffer on cellular intake of recombinant protein. Western blotting analysis of whole cell lysates from cells in G.

**Figure 1—figure supplement 3.**
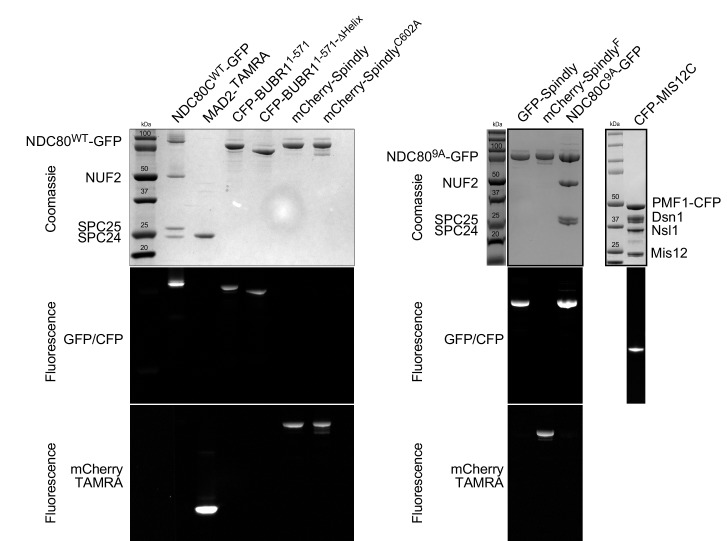
Recombinant proteins used for this study. Protein samples employed in this study were run on an acrylamide gel and imaged by coomassie staining or for the indicated fluorophore. Note: the gel representing fluorescent CFP-MIS12 was run with a sample that was not boiled (to preserve fluorescence) and this resulted in a faster migration.

**Figure 1—figure supplement 4.**
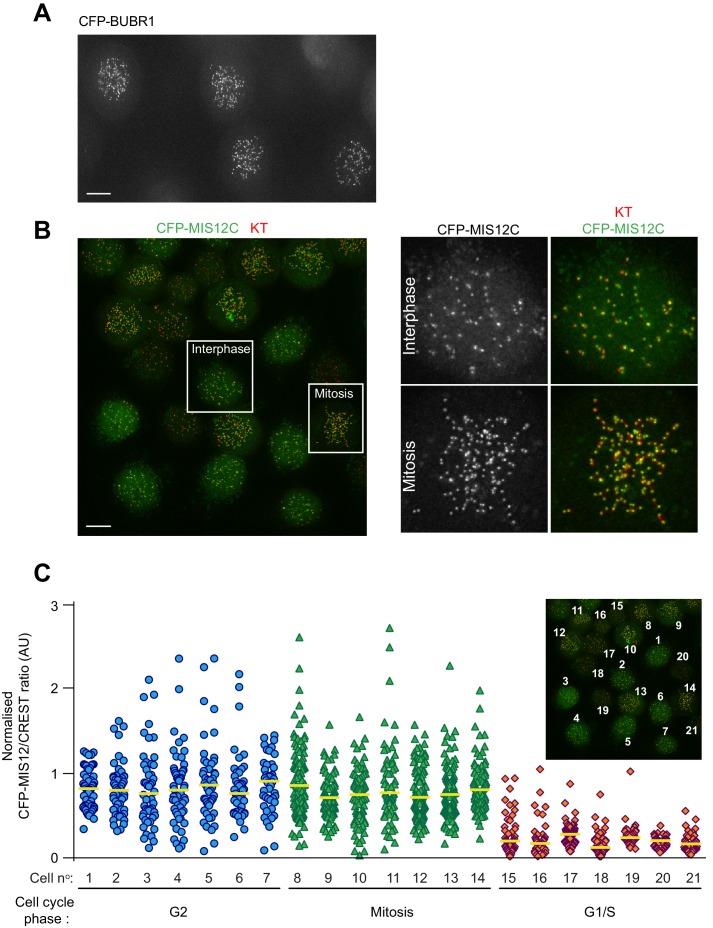
EP is highly efficient and produces uniform protein levels between different recipient cells. (**A**) representative imaging field shows HeLa cells electroporated with either CFP-BUBR1 (**A**) or CFP-MIS12C (**B**) and, after recovery, treated with nocodazole for 4 hr. Insets in B represent magnification of the indicated cells. KTs were stained with the marker CREST. Scale bar = 5 μm. (**C**) Quantification of KT levels for electroporated CFP-MIS12C from cells in panel B. Individual cell numbers are indicated in the small inset. Cell phases were assigned based on cell morphology and size. Each symbol represents a single KT. Yellow lines indicate median.

**Figure 1—figure supplement 5.**
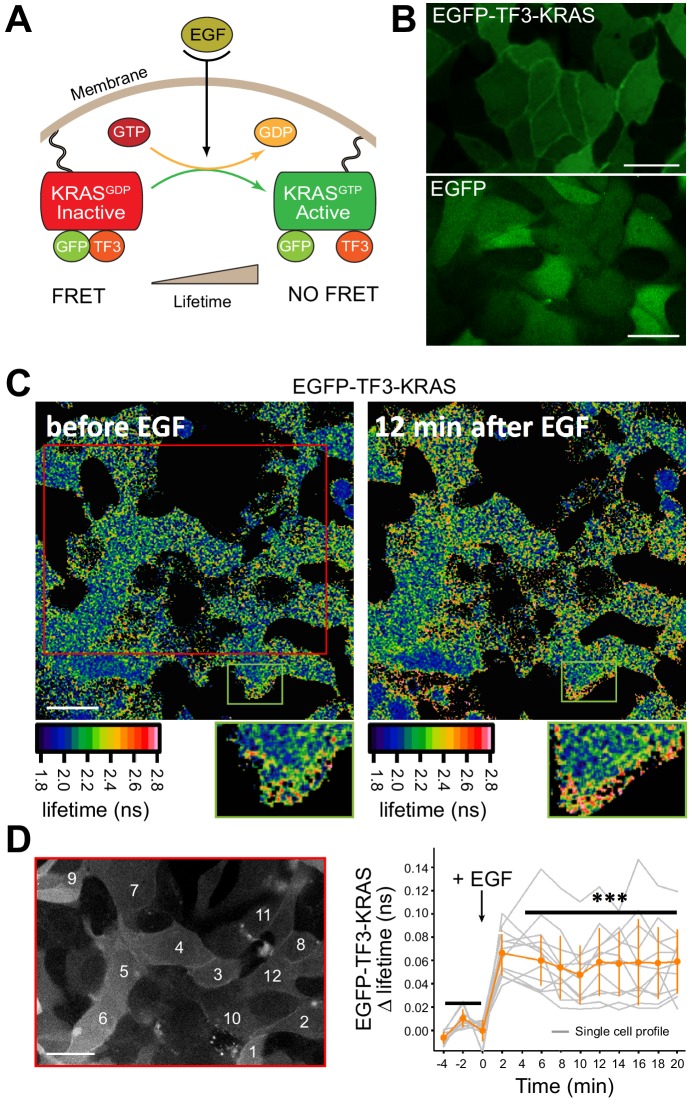
Live-cell FLIM-FRET on EP-delivered proteins. (**A**) Schematic representation of the activation of the membrane-bound EGFP-TF3-KRAS FRET-sensor. Treatment with EGF results in a conformational change which results in a loss of FRET between the fluorescent moieties of the sensor and an increase in the donor fluorescence lifetime measured by FLIM-FRET live microscopy. (**B**) Membrane localization of EP-delivered EGFP-TF3-KRAS in serum-starved MDCK interphase cells. Scale bar = 5 μm. (**C**) Analysis of spatiotemporal dynamics of electroporated EGFP-TF3-KRAS signaling by live-cell FLIM-FRET imaging. Upon EGF stimulation, the conformational switch in intracellular EGFP-TF3-KRAS results in loss of FRET signal and a subsequent increase in the lifetime of the GFP-donor. Lifetime values for each pixel are shown color-coded. Scale bar = 5 μm. (**D**) Activity of electroporated EGFP-TF3-KRAS is qualitatively and quantitatively identical to the one obtained in EGFP-TF3-KRAS microinjection experiments (Voss et al., 2016). Graph shows single cell readouts of fluorescence lifetime profiles. Gray traces represent individual measurements while orange line represents the mean. Error bars are SDM. Scale bar = 5 μm.

The advantage of using kinetochores in this study is that they form small, discrete cellular structures with a very recognizable punctuate pattern and several markers are available to probe their native composition, thus making it very easy to assess correct localization of exogenously introduced variants. Therefore, thecorrect assembly of kinetochore complexes upon subunit delivery by EP can be inspected at different levels of structural and functional resolution (Musacchio and Desai, 2017). Here, we report the suitability of electroporation to deliver individual kinetochore proteins into different mammalian cell types and their correct assembly into native kinetochore complexes. We further show that electroporation can be used to transduce pre-assembled kinetochore components to functionally complement depleted KT subunits. Collectively, our results provide very encouraging evidence that EP can be used to deliver recombinant proteins of interest, even when chemically modified, into target cells for functional complementation in a non-invasive manner.

## Results and discussion

### Electroporation: an outline

As outlined in Figure 1B and as described in detail in the Methods section, intracellular protein delivery by EP is a simple procedure that does not require specialized handling skills or dedicated instrumental set-ups, making it easy to establish in any laboratory. Briefly, in preparation for EP, cells can be treated under various experimental regimes, for example for cell cycle synchronization (Ciossani et al., 2018), before being harvested, washed, and resuspended in an electroporation buffer containing the diluted protein (the ‘EP slurry’). The EP slurry is subjected to multiple electric pulses that create reversible ruptures in the cell’s membrane and allow intracellular protein delivery. Following EP, we subjected cells to mild trypsinization to remove non-internalized, membrane-associated proteins and then allowed cells to recover, typically between 16 and 18 hr, but earlier times are possible (e.g. 4 hr), before being prepared for the desired analysis, such as live imaging, immunofluorescence, immunoprecipitation or western blotting experiments. Using model recombinant protein like α-Synuclein (made fluorescent by labeling with the synthetic dye Atto488) or mCherry, which can reach very high concentrations, we determined that intracellular uptake scaled linearly with input concentrations irrespective of the manipulated cell types (Figure 1—figure supplement 1A–B). These results suggested that EP maybe broadly applicable to transduce defined amounts of recombinant proteins into cultured mammalian cells, with individual cells harboring uniform concentrations of exogenously delivered proteins (Figure 1—figure supplement 1C–D). Importantly, EP is efficient across multiple cell lines and different electroporation conditions, and treated cells usually showed limited damage or toxicity (Figure 1—figure supplement 2A–B). However, the post EP recovery is important to restore normal cell physiology and morphology and to allow for adhesion to be re-established (if required). The latter may require at least four hours. EP parameters used for delivery, such as composition of the EP buffer or settings of the EP pulse (voltage strength, duration, and number of repetitions) are specific for each different electroporation system and must be optimized accordingly. Because the electroporation systems used for this study is proprietary, some of the electroporation settings are not known (e.g EP buffer composition). A comparison of the effects of different parameters on electroporation efficiency is presented in Figure 1—figure supplement 2C–H.

Previously, we generated various recombinant versions of stable sub-complexes of the human kinetochore, and demonstrated that they could be used to reconstitute large parts of the kinetochore and of the spindle assembly checkpoint (SAC) in vitro (Huis In 't Veld et al., 2016; Faesen et al., 2017; Weir et al., 2016; Pesenti et al., 2018). This library of high-quality recombinant samples (Figure 1—figure supplement 3) already extensively characterized in vitro represented an excellent resource to address the general suitability of EP for in vivo delivery and functional analysis. We initially tested this approach on BUBR1 (BUB1-related), a multi-domain protein that operates in the SAC. Recombinant BUBR1^1-571^ (encompassing residues 1–571 of the 1050-residue BUBR1 protein, which are sufficient for checkpoint function) (Faesen et al., 2017) fused to a cyan fluorescent protein (CFP) was introduced into HeLa cells at an EP slurry concentration of 10 μM. In mitotically arrested cells (by addition of the spindle poison nocodazole, a condition that activates the SAC), CFP-BUBR1^1-571^ distinctly localized to kinetochores, as revealed by the typical punctuate staining and by co-localization with CREST, an established kinetochore marker (Figure 1C). We have shown previously that BUBR1 kinetochore recruitment in human cells requires dimerization with kinetochore-bound BUB1, a BUBR1 paralog, through segments that encompass a predicted helical domain in the two proteins (Overlack et al., 2015). In agreement with these previous observations, recombinant CFP-BUBR1^1-571-∆Helix^, a deletion mutant of BUBR1 lacking the BUB1 binding helix (deletion of residues 432–484) was unable to localize to kinetochores (Figure 1D–F). These results demonstrated that EP delivery of functional CFP-BUBR1^1-571^ resulted in kinetochore localization, whereas transduction of a KT binding-compromised mutant of BUBR1 did not. The efficiency of CFP-BUBR1^1-571^ delivery was high and most cells displayed uniform levels of the recombinant protein (Figure 1—figure supplement 4A). Thus, electroporation can deliver very similar amounts of recombinant protein into each cell. In the course of this study, this became to be recognized as a general feature of this approach, largely independently of the protein used.

We used the Sortase-ligation method (Popp et al., 2009) to fuse a fluorescent peptide modified with the dye tetra-methyl-rhodamine (TAMRA) to the C-terminus of MAD2, another SAC protein. The resulting MAD2^TAMRA^ was delivered by EP in HeLa cells at an EP-slurry concentration of 5 μM. Like CFP-BUBR1^1-571^, MAD2^TAMRA^ was found to localize effectively at kinetochores of nocodazole-treated cells, as shown by co-localization with CREST (Figure 1G). Furthermore, we performed co-delivery of CFP-BUBR1^1-571^ with MAD2^TAMRA^, and found both proteins at unattached KTs in nocodazole-arrested cells (Figure 1H). Thus, individual or multiple fluorophore-tagged components of the human KT are faithfully targeted to their respective binding sites after EP.

Besides protein localization, we asked whether EP-delivery of fluorescently labeled samples can be exploited for spectroscopic applications such as FLIM-FRET microscopy (Bastiaens and Squire, 1999), to study the spatiotemporal dynamics of proteins in cells. To this end, we focused on KRAS/p21, a small, globular GTPase with important regulatory functions in many signaling pathways (Schmick et al., 2014). We studied KRAS signaling in live cells electroporated with GFP-tagged and Tide Fluor 3 (TF3)-labeled human KRAS (i.e. EGFP-TF3-KRAS, final size ~50 kDa), a conformational sensor for GTPase activity (COSGA) obtained by chemical labeling of recombinant protein (Voss et al., 2016) (Figure 1—figure supplement 5A). Activation and conformational switching of EGFP-TF3-KRAS occurs upon epidermal growth factor (EGF) stimulation and causes the spatial separation of the fluorescence moieties and a concomitant loss of intramolecular FRET signal, which ultimately results in an increase of the donor fluorescence lifetime measured by FLIM-FRET microscopy (Voss et al., 2016). Upon EP-delivery, we found that EGFP-TF3-KRAS, but not EGFP, predominantly localize to the plasma membrane (Figure 1—figure supplement 5B), with a pattern indistinguishable from endogenous, over-expressed, or microinjected KRAS (Voss et al., 2016; Schmick et al., 2014; Schmick et al., 2015). Following addition of EGF, we observed the predicted increase in the donor lifetime of EGFP-TF3-KRAS (Figure 1—figure supplement 5C–D). Importantly, our EP-based results were qualitatively and quantitatively identical to the ones that were observed in EGFP-TF3-KRAS microinjection experiments (Voss et al., 2016), demonstrating the suitability of EP for FRET-based assays in living cells as a valid alternative to protein delivery by microinjection.

### Electroporated MIS12 complex targets kinetochores and functionally complements depletion of the endogenous complex

The experiments discussed in the previous section described the delivery of single polypeptides. Next, we tested the suitability of EP for the delivery of multi-subunit protein complexes. To this end, we started with the Mis12 complex (MIS12C, where ‘C’ stands for ‘complex’), an extremely stable four-subunit kinetochore assembly of the DSN1, NSL1, MIS12, and PMF1 subunits with a molecular mass of ~120 kDa. In previous studies, we demonstrated that the stability of the MIS12C requires co-expression of all four constituent subunits and reported the development of various co-expression vectors for its heterologous reconstitution in insect cells (Petrovic et al., 2016; Petrovic et al., 2014; Petrovic et al., 2010). Here, we generated a fluorescence version of MIS12C by fusing the coding sequence of cyan fluorescent protein (CFP) to that of the PMF1 subunit. We then co-expressed the subunits in insect cells and purified the resulting complex (CFP-MIS12C) to homogeneity (Figure 1—figure supplement 3).

We electroporated CFP-MIS12C into cycling HeLa cells and monitored CFP-MIS12C levels by fluorescent microscopy. At an EP slurry concentration of 8 μM, CFP-MIS12C was clearly visible at kinetochores in the large majority of electroporated cells and with remarkably similar kinetochore levels within the same cell and between different cells (Figure 1—figure supplement 4B and C). These findings further indicated that assembled CFP-MIS12C remained intact during the electroporation procedure and reached the cytoplasm in a kinetochore-binding competent, functional state.

**Figure 2.**
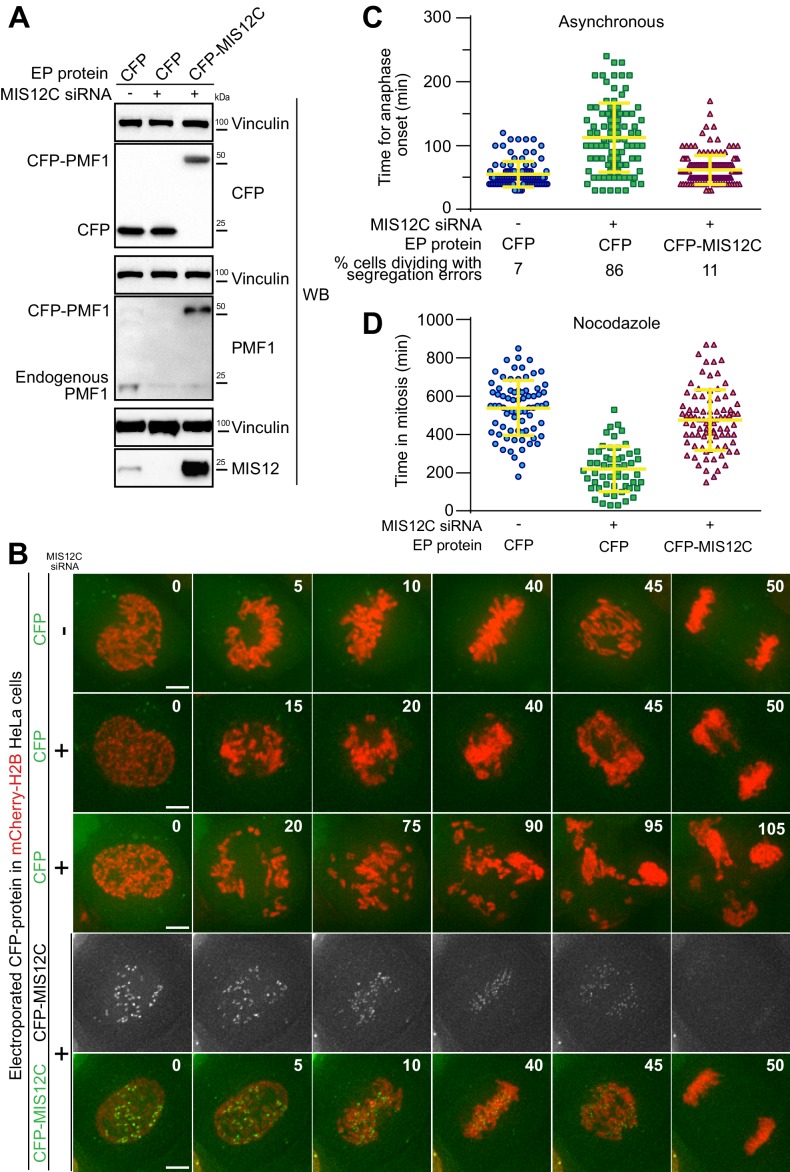
Electroporated MIS12 complex targets kinetochores and functionally complements depletion of the endogenous complex. (**A**) The indicated proteins, including CFP-labeled recombinant MIS12C, were electroporated in mCherry-H2B-expressing HeLa cells previously treated with siRNAs to deplete the endogenous MIS12C. Western blotting of cellular protein levels in extracts was performed and showed a depletion in MIS12 and PMF1 levels of 93,7% and 87,6% respectively. (**B**) Frames collected at the indicated time points (min) of time-lapse live-cell fluorescence microscopy movies depict chromosome congression and segregation of cells from A. Two representative phenotypes observed in cells with depleted MIS12C that were concomitantly electroporated with CFP as control are shown. Green and red signal represent, respectively, CFP proteins and DNA. Scale bar = 5 µm. (**C**) Quantification of chromosome segregation timing and defects in cells from A-B. Each symbol represents a measure of a single cell mitotic duration until anaphase onset (based on DNA and cell morphology). Yellow lines indicate mean values ± SD. (**D**) Electroporated CFP-MIS12C rescues SAC defects in H2B-mCherry HeLa cells depleted for endogenous MIS12C. Duration of mitosis (based on DNA and cell morphology) of individual cells treated as in A but imaged in the presence of nocodazole 0.3 μM. Yellow lines indicate mean values ± SD.

In a more stringent test, we also asked if CFP-MIS12C retained its expected kinetochore function after electroporation. MIS12C is as an assembly hub for the kinetochore, being responsible for the recruitment of a subset of the microtubule-binding NDC80 complexes as well as of KNL1, a signaling platform for the SAC (Petrovic et al., 2010; Kiyomitsu et al., 2011) (Figure 1A). These functions make MIS12C necessary for timely chromosome congression and faithful cell division. To test the functionality of the recombinant CFP-MIS12 complex, we performed a complementation assay in HeLa cells depleted of endogenous MIS12 complex by the RNA interference (RNAi) methods using silencing RNAs (siRNAs) against multiple subunits, as previously described (Kim and Yu, 2015). Effective depletion of MIS12C subunits and uptake of fluorescent proteins was demonstrated by western blotting (Figure 2A). This is noteworthy, as it exposes a fundamental advantage of EP over another protein targeting technique such as microinjection, which is limited to a small number of target cells and therefore unsuitable to biochemical analyses. In agreement with previous reports, depletion of MIS12C compromised chromosome congression (Figure 2B–C), a consequence of reduced kinetochore levels of NDC80 and KNL1 (Kim and Yu, 2015). As shown by live-cell microscopy, EP delivery of CFP-MIS12C into depleted cells, but not of a CFP control, rescued chromosome congression essentially to the levels observed in control cells, indicating that the recombinant complex is functionally active (Figure 2B–C and Videos 1–4). Complete functional rescue of MIS12 activity appeared to require somewhat higher intracellular levels of recombinant MIS12 than those of the endogenous counterpart (Figure 2—figure supplement 1). The reasons for this are unclear, but may include limited proteolysis of residues relevant for MIS12 function and localization, or, alternatively, a rate-limiting addition of important post-translational modifications.

**Figure 2—figure supplement 1.**
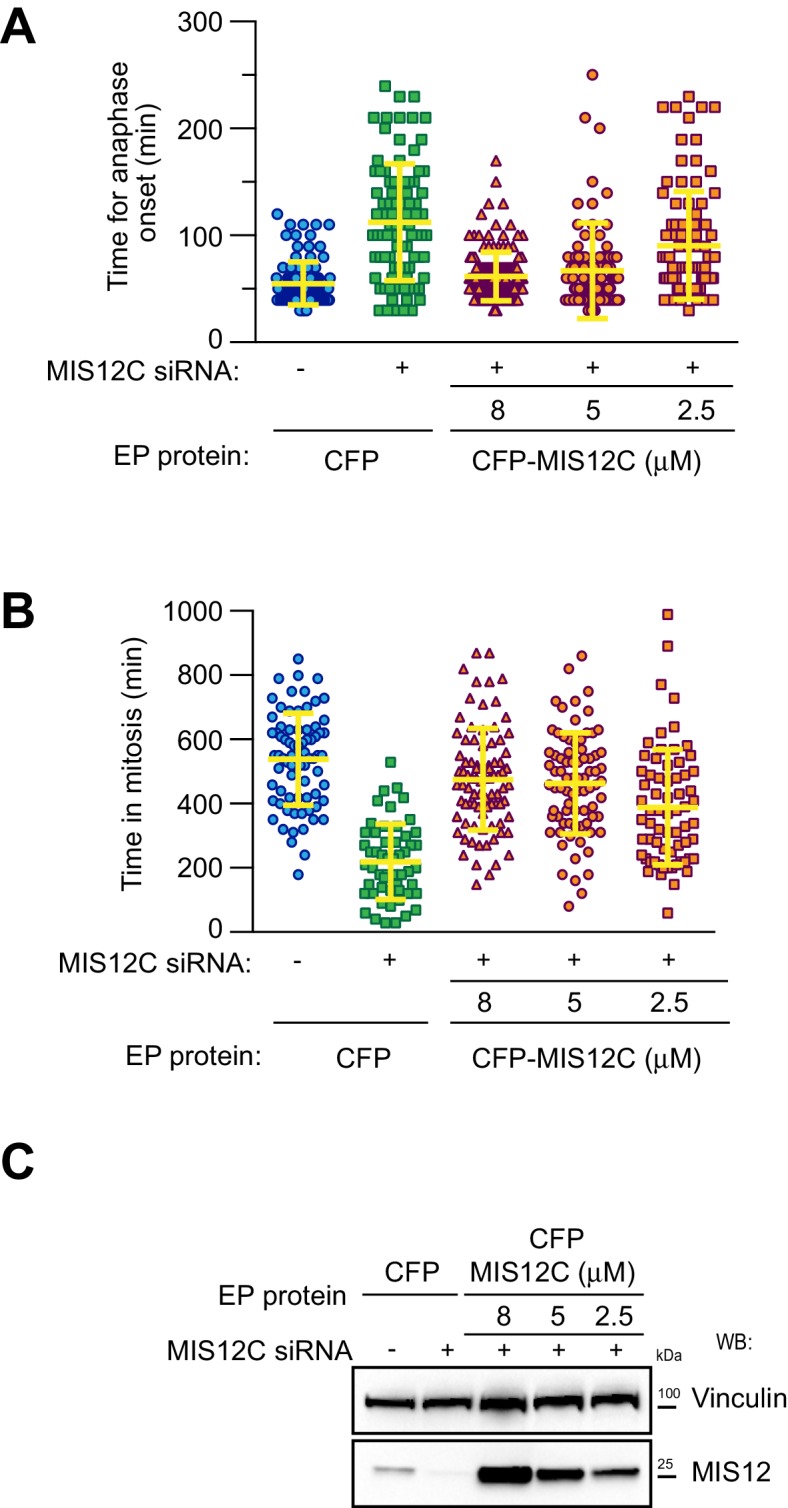
Electroporation of MIS12 complex at different EP-slurry concentrations. (**A**) Quantification of chromosome segregation timing in H2B-mCherry HeLa cells depleted for endogenous MIS12C and electroporated with decreasing concentrations of recombinant CFP-MIS12C in the EP-slurry. Each symbol represents a measure of a single cell mitotic duration until anaphase onset (based on DNA and cell morphology). Yellow lines indicate mean values ± SD. Values indicated for Control/CFP, siRNA/CFP and siRNA/CFP-MIS12C 8 μM are the same shown in Figure 2C. (**B**) Duration of mitosis (based on DNA and cell morphology) of H2B-mCherry HeLa cells treated as in A but imaged in the presence of nocodazole 0.3 μM. Yellow lines indicate mean values ± SD. Values indicated for Control/CFP, siRNA/CFP and siRNA/CFP-MIS12C 8 μM are the same shown in Figure 2D. (**C**) Western blotting of MIS12 cellular levels in extracts from A-B.

**Video 1.** Live imaging movie of the cell shown in Figure 2B (Ctrl+CFP). Images show GFP-signal in green and DNA signal in red.

**Video 2.** Live imaging movie of the cell shown in Figure 2B (siRNA+CFP example 1).

**Video 3.** Live imaging movie of the cell shown in Figure 2B (siRNA +CFP example 2).

**Video 4.** Live imaging movie of the cell shown in Figure 2B (siRNA +CFP-MIS12C).

Improperly attached kinetochores trigger the spindle assembly checkpoint (SAC), causing a mitotic arrest that delays entry into anaphase (Musacchio, 2015). The checkpoint pathway exhausts its function only after all chromosomes have achieved bi-orientation on the mitotic spindle. Despite causing dramatic chromosome congression failures, depletion of endogenous MIS12C is hardly compatible with checkpoint function because cells lacking the MIS12C also fail to recruit KNL1, which is required for SAC signaling. Thus, MIS12C-depleted cells have a strongly weakened SAC and enter anaphase prematurely (Hara et al., 2018; Kim and Yu, 2015). To assess SAC function under our siRNA conditions, we treated cells with 0.3 μM nocodazole, a microtubule-depolymerizer, thus creating conditions that potently activate checkpoint signaling in control cells. We then measured, by live-cell video microscopy, the cells’ ability to sustain a prolonged mitotic arrest. In agreement with the previous studies, cells depleted of MIS12C were unable to mount a strong mitotic arrest and underwent mitotic exit in the presence of multiple unattached or incompletely attached chromosomes, indicative of a checkpoint defect. Electroporated CFP was unable to restore the SAC response, while recombinant CFP-MIS12C restored mitotic duration to values similar to those observed in control cells, indicating a full rescue of SAC activity (Figure 2D). Collectively, these results indicate that electroporated recombinant MIS12C targeted the KTs as a functionally intact complex and that EP can be used for biological complementation assays in combination with siRNA-mediated knockdown routines.

### EP of a protein complex with an exceptionally large hydrodynamic radius and its interaction with endogenous partners

**Figure 3.**
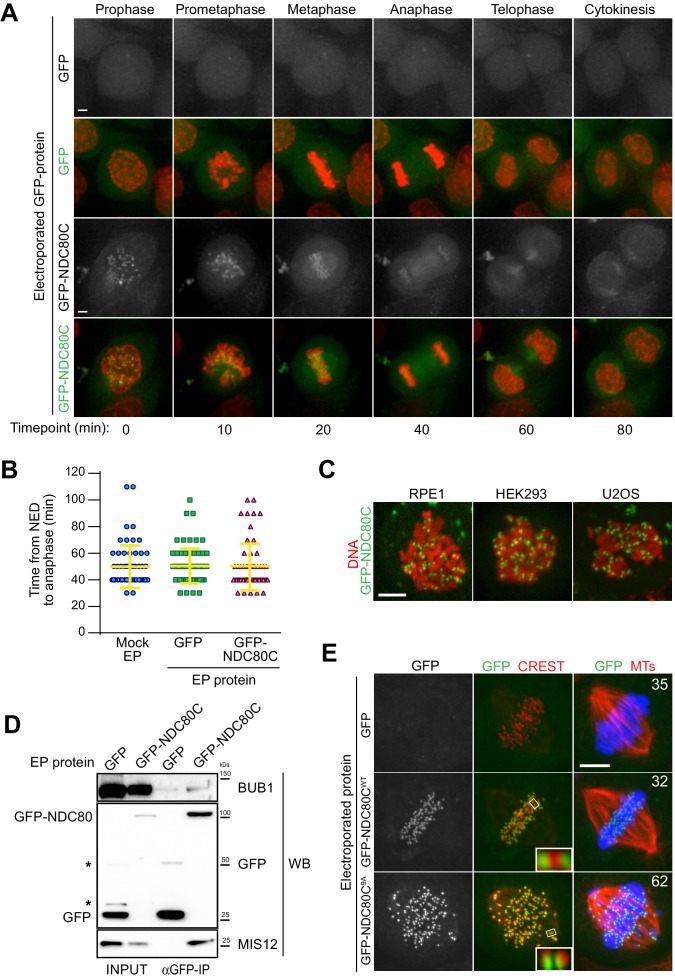
Cellular delivery of a protein complex with an exceptionally large hydrodynamic radius. (**A**) GFP-labeled recombinant NDC80C^WT^ binds to kinetochores and its localization is compatible with normal mitotic progression. Panels show timepoints of time-lapse movies for asynchronously growing HeLa cells electroporated with recombinant GFP proteins and imaged live every 10 min. DNA was stained with SiR-Hoechst-647 and is shown in red. Scale bar = 5 µm. (**B**) Quantification of time periods until anaphase onset for each of the conditions in panel A. Each symbol represents a single cell. Yellow lines indicate mean values ± SD. (**C**) Additional human cell lines were electroporated with GFP-NDC80C. RPE1 cells are non-transformed human retinal pigmented epithelium cells. HEK293 cells are human epithelium kidney cells. U2OS cells are human osteosarcoma cells. Following recovery and staining with SiR-Hoechst-647 DNA dye, cells were imaged live and display a clearly visible kinetochore localization of the delivered GFP-NDC80C complex. Scale bar = 5 µm. (**D**) Immunoprecipitation analysis of protein extracts from cells treated as in A shows that EP-delivered GFP-NDC80C^WT^ establishes normal interactions with its endogenous KT partners. Western blotting panels show immunoprecipitates performed with α-GFP beads and probed with the indicated antibodies. (**E**) An error correction assay for the EP-delivery of a GFP-NDC80C^9A^ mutant shows a dominant-negative effect on the correction of improper KT-MT attachments. Panels display representative images of electroporated HeLa cells treated with STLC to accumulate erroneous synthelic KT-MT attachments, followed by STLC washout, release into MG132 (proteasome inhibitor that prevents mitotic exit), and fixation 150 min after STLC-release. Inability to correct erroneous attachments results in uncongressed chromosomes. Control cells were electroporated with GFP alone. Numbers represent the percentage of cells with uncongressed chromosomes for each condition. KTs were labeled with anti-CREST immunostaining, microtubules (MT) with an anti-TUBULIN antibody and DNA with DAPI. Insets represent magnifications of the indicated KT. Scale bar = 5 µm.

With a long axis of approximately 20 nm (Petrovic et al., 2016; Petrovic et al., 2014) the MIS12C is a rather elongated molecule. Despite these features, intracellular delivery by EP was highly efficient and did not appear to be size limited. To further investigate possible limitations for EP-mediated delivery of biological assemblies with similar dimensions, we chose the four subunit NDC80C complex as our next target. With its dumbbell shape consisting of a coiled-coil shaft flanked by terminal globular domains, the four-subunit NDC80C complex reaches approximately ~60 nm in length (Huis In 't Veld et al., 2016; Wei et al., 2005). We were curious to assess if extreme elongation of NDC80C affected EP efficiency. As for the MIS12C, also the ~180 kDa NDC80C achieves stability through co-expression of its four subunits (NUF2, SPC24, SPC25, and NDC80) in insect cells (Petrovic et al., 2014; Wei et al., 2005; Figure 1A). After expression and purification of a GFP-tagged version of NDC80 from insect cells (Figure 1—figure supplement 3), we electroporated GFP-NDC80 into HeLa cells at a final concentration of 5 µM. 12 hr after electroporation, cells were imaged by live cell video-microscopy. This revealed kinetochore localization of the recombinant GFP-NDC80 complex (Figure 3A), indicating that the considerable length of the complex is compatible with efficient delivery.

Manipulated HeLa cells carrying recombinant NDC80C-GFP in addition to endogenous NDC80C displayed no alterations in cell cycle progression and proceeded through the different stages of mitosis without detectable changes when compared to control cells (Figure 3A–B and Videos 5–9). Thus, delivering exogenous GFP-NDC80C does not cause obvious cell cycle alterations or cytotoxicity. We reached similar conclusions when we delivered GFP-NDC80C into human RPE, HEK293 and U2OS cells (Figure 3C).

**Video 5.** Live-imaging of asynchronous HeLa cells electroporated with recombinant GFP. Fluorescence time-lapse images show GFP signal (left panel), DNA signal (central panel) and a merge (right panel).

**Video 6.** Live-imaging of asynchronous HeLa cells electroporated with GFP-NDC80C.

**Video 7.** Live-imaging movie of cells shown in Figure 3A–B (Mock). Images show GFP-signal in green and DNA signal in red.

**Video 8.** Live-imaging movie of cells shown in Figure 3A-B (GFP).

**Video 9.** Live-imaging movie of cells shown in Figure 3A–B (GFP-NDC80C).

To test whether recombinant GFP-NDC80C- complexes establish their expected physical interactions after EP delivery, we performed GFP-immunoprecipitation (GFP-IP) assays on protein extracts generated from mitotic HeLa cells previously electroporated with GFP-NDC80C. Western blotting demonstrated that MIS12 and BUB1 were present in the NDC80C-GFP precipitates, but not in the precipitates of GFP (Figure 3D). Thus, the electroporated GFP-NDC80C establishes physiologic interactions with its endogenous binding partners, demonstrating the versatility of EP as a technique to probe protein interactions in living cells both through imaging and through biochemistry.

### An NDC80C mutant triggers a dominant-negative chromosome alignment defect

Chromosome bi-orientation implies that the sister kinetochores attach to microtubules emanating from opposite spindle poles. Incorrect attachments that occur during mitosis are sensed by an error correction mechanism that, by regulating the phosphorylation state of the unstructured N-terminal region of the NDC80 subunit, causes the destabilization of incorrect attachment and the formation of new, correct ones (Monda and Cheeseman, 2018). A previously described NDC80 mutant (9A) carrying nine alanine mutations at the phosphorylation sites in the protein’s N-terminus establishes hyper-stable KT-MT attachments that cannot be corrected, resulting in frequent chromosome congression errors (Sundin et al., 2011; Etemad et al., 2015; Tauchman et al., 2015). We asked if a GFP-NDC80C^9A^ mutant electroporated in HeLa cells had dominant-negative effects in an established biorientation and error correction assay (Lampson et al., 2004). HeLa cells were allowed to enter mitosis in presence of STLC, an inhibitor of the Eg5 kinesin (Skoufias et al., 2006) whose activity is crucially required for centrosome separation and spindle bipolarization, resulting in the accumulation of monopolar spindles in which sister kinetochores frequently attach to the same microtubule organizing center (MTOC, the centrosome in this case) in a condition known as syntelic attachment. Upon washout of STLC, Eg5 reactivation, and spindle bipolarization, a functional error correction pathway is required to resolve the syntelic attachments and achieve correct metaphase alignment (Lampson et al., 2004). In this setup, control cells electroporated with GFP or GFP-NDC80C^WT^ corrected erroneous attachments and aligned their chromosomes properly at the metaphase plate (Figure 3E). In contrast, cells electroporated with the GFP-NDC80C^9A^ mutant were impaired in their correction pathway, and accumulated misaligned chromosomes with high frequency. Based on these findings, we concluded that EP-delivered GFP-NDC80C^9A^ exerted a dominant-negative effect over endogenous NDC80C in its ability to correct and repair chromosome misalignments in response to STLC treatment.

### In vitro farnesylation allows spindly localization when Farnesyl Transferase is inhibited

Spindly is an adaptor protein that promotes the interaction of the minus-end-directed motor Dynein with its processivity factor Dynactin (Griffis et al., 2007; Gassmann et al., 2008). Spindly also interacts directly with the ROD-Zwilch-ZW10 (RZZ) complex, the main constituent of the so-called kinetochore corona, a crescent-shaped structure that assembles on kinetochores in early prometaphase to promote microtubule capture (Magidson et al., 2015) (Figure 1A). Kinetochore localization of Spindly critically depends on its ability to interact with the RZZ complex (Holland et al., 2015; Moudgil et al., 2015). The latter, in turn, requires the irreversible post-translational isoprenylation of Spindly with a farnesyl moiety on a cysteine residue near the Spindly C-terminus (Holland et al., 2015; Moudgil et al., 2015; Mosalaganti et al., 2017). Farnesylation is carried out by the enzyme farnesyl-transferase (FT), which, in addition to Spindly, also targets several members of a family of Ras-like small GTP binding proteins, including Ras itself (Appels et al., 2005). Potent FT inhibitors have been described, and previous studies demonstrated that the localization of Spindly to kinetochores is prevented when FT is inhibited (Holland et al., 2015; Moudgil et al., 2015).

**Figure 4.**
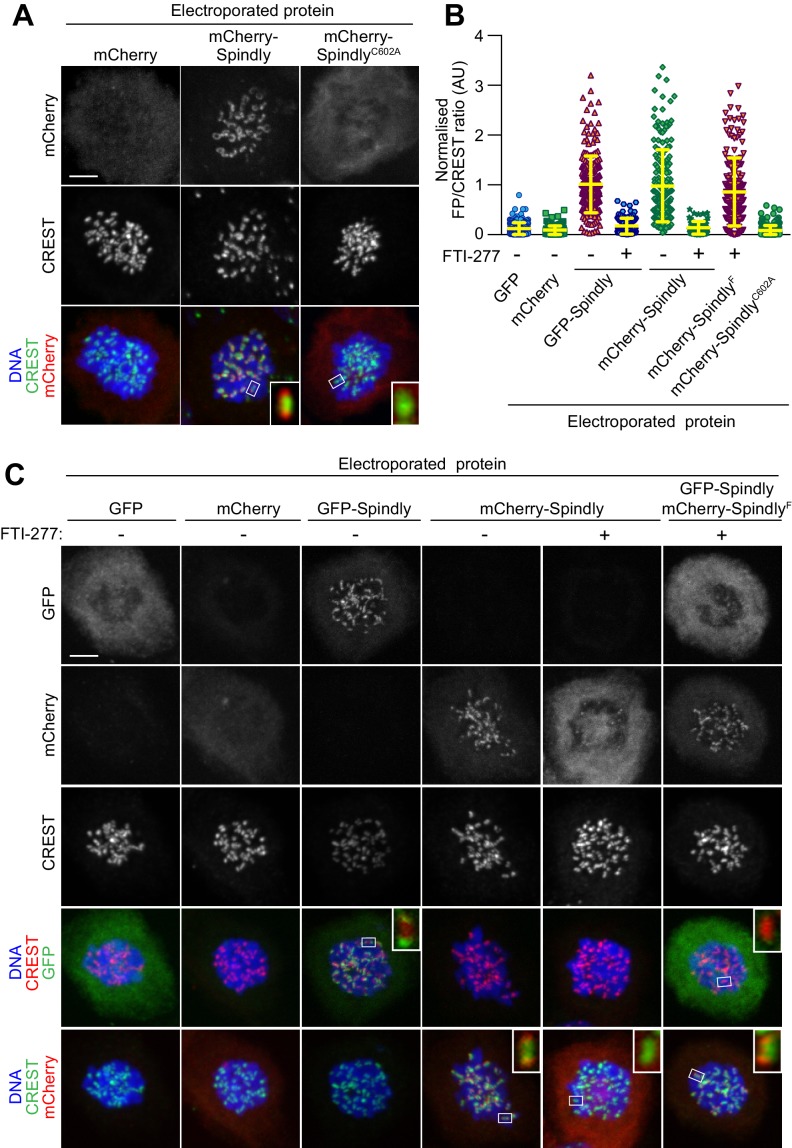
In vitro farnesylation allows Spindly localization when farnesyl transferase is inhibited. (**A**) Kinetochore localization of unmodified recombinant Spindly depends on cellular farnesylation. Panels show representative images of cells transduced with unfarnesylated mCherry-Spindly in nocodazole-treated HeLa cells. Mutation of Cys602 to alanine prevents farnesylation upon EP and reduces KT levels of the delivered protein. Kinetochores were stained with CREST antibodies and DNA with DAPI. Controls cells were electroporated with mCherry. Insets represent magnifications of the indicated kinetochores. Scale bar = 5 µm. (**B**) Quantification of KT levels for electroporated Spindly from cells in A and C. Each symbol represents a single cell. Yellow lines indicate mean values ± SD. (**C**) In vitro farnesylation of mCherry-Spindly before EP bypasses the need of cellular farnesylation to achieve kinetochore localization. Cells treated with the farnesyl-transferase inhibitor FTI-277 were electroporated with either GFP, GFP-Spindly, mCherry, mCherry-Spindly, or in vitro pre-farnesylated mCherry-Spindly (mCherry-Spindly^F^) and then processed for immunofluorescence analysis. Kinetochores were stained for CREST and DNA with DAPI. Insets represent magnifications of the indicated KT. FP = Fluorescent protein. Scale bar = 5 µm.

Because farnesylation of Spindly has been reconstituted in vitro with purified components (Mosalaganti et al., 2017), we reasoned that we could exploit this reaction to promote kinetochore localization of recombinant Spindly in cells experiencing a long-term blockade of FT activity. Two versions of HsSpindly that were N-terminally fused to GFP or mCherry (molecular mass ~95 kDa) were expressed in insect cells and purified to homogeneity (Figure 1—figure supplement 3). Our previous mass spectrometry analysis demonstrated that only trace farnesylation of Spindly takes place in insect cells (Mosalaganti et al., 2017). When we EP-delivered fluorescent recombinant mCherry-Spindly into HeLa cells, we detected a fluorescent signal at the periphery of kinetochores that was comparable to the localization described for endogenous Spindly (Figure 4A–B) (Holland et al., 2015), or of Spindly introduced by microinjection (unpublished observation). mCherry-Spindly^C602A^, a mutant that cannot be farnesylated, failed to show any KT localization upon EP (Figure 4A–B). This indicated that wild type mCherry-Spindly becomes farnesylated after intracellular delivery. To further substantiate this, we treated cells with FTI-227, a selective farnesyl-transferase inhibitor (Appels et al., 2005). In the presence of FTI-227, localization of recombinant mCherry-Spindly or GFP-Spindly was greatly reduced (Figure 4B–C), confirming that FT activity is required for Spindly localization.

We therefore asked if in vitro farnesylation by FT rescued the localization of Spindly in cells treated with FTI-227. mCherry-Spindly was farnesylated in vitro with recombinant farnesyltransferase (FT) and farnesyl-pyrophosphate as a substrate, as described previously (Mosalaganti et al., 2017). The resulting farnesylated mCherry-Spindly (mCherry-Spindly^F^) and untreated GFP-Spindly were co-electroporated at the same EP slurry concentration (10 µM) in HeLa cells that had been treated with FTI-227 for the previous 24 hr. Remarkably, mCherry-Spindly^F^, but not GFP-Spindly, successfully targeted kinetochores, showing that the complementation of FT activity in vitro had bypassed the blockade of FT activity in cells by FTI-227. Although we do not provide formal proof, the persistent treatment with FT inhibitors in this experiment suggests that Spindly^F^ may be the only residual pervasively farnesylated protein in the target cells. Together, these findings substantiated the notion that pre-farnesylation of recombinant Spindly bypassed the need for endogenous farnesyl-transferase activity for proper kinetochore localization in HeLa cells.

### Conclusions

The modification of biological macromolecules through the introduction of new functionalities has progressed at a tremendous pace in recent years, enriching the palette of tools for biological investigation with a wide spectrum of functionalities. For instance, genetic code expansion, an approach that allows to introduce chemical functionalities into proteins, ranging from synthetic, photostable fluorescent dyes with high quantum yields to reactive groups (e.g. UV-activated cross-linkers and click chemistry handles), is undergoing an explosive development (Davis and Chin, 2012; Nikić and Lemke, 2015). With rapid growth of chemical and synthetic biology, the demand for robust methods to deliver modified synthetic or semi-synthetic macromolecules into cells is growing (Lienert et al., 2014).

While electroporation has been long recognized for its potential as a delivery approach, questions have been raised regarding the degree of its invasiveness and damage inferred on cellular structures, most notably membranes (Torchilin, 2008). Here, we have assessed the suitability of batch EP for delivering recombinant proteins into living mammalian cells for various applications in cell biology. In comparison to previous studies that focused on cytosolic proteins devoid of specific localization, our focus was on kinetochores, which are small, discrete subcellular structures. This was crucial, because it enabled a detailed assessment of the effective degree of functional complementation of the electroporated samples. Our results identify EP as a rapid, efficient, and semi-quantitative technique that enables the delivery into various cultured mammalian cell lines of proteins of variable mass and hydrodynamic radius, including the highly elongated NDC80C. Importantly, we provide clear evidence that the delivered proteins remain largely structurally and functionally intact after delivery, as witnessed by their correct kinetochore localization and, where applicable, ability to complement siRNA-based depletion, dominant negative effects, and immunoprecipitation with endogenous interacting partners. Thus, in addition to its ease of application, EP is very attractive because it allows delivery in sufficiently large cohorts of cells, and is therefore compatible with cell biochemistry, as clearly shown here.

We envision EP to be generally compatible with many macromolecular interaction approaches, from mass spectrometry to immunoprecipitation. If combined with suitable chemical modifications of the probes, for instance by introduction of acutely activatable crosslinking groups, EP may become a method of choice for the identification of elusive binding partners (for instance, low affinity substrates of enzymes) with high spatial and temporal resolution. Here, we have demonstrated how the introduction of a farnesyl chain in vitro rescued the kinetochore localization of Spindly in cells treated with a farnesyl transferase inhibitor. In the future, this approach may be extended to other lipid modifications, and may allow monitoring the behavior of only one or a few modified proteins at the time. Another obvious advantage of in vitro manipulations of macromolecules is that it allows precision labeling with small fluorescent dyes, potentially bypassing limits associated with tagging target proteins with bulky genetically encoded fluorescent proteins. In this context, EP would expand the toolbox of reagents suitable for live-cell spectroscopic applications such as FLIM-FRET microscopy or FRAP analysis. In summary, we envision that EP has the potential to become a method of choice for delivery of synthetic or semi-synthetic proteins into the cellular environment.

## Materials and methods

**Key resources table:** 

Reagent type (species) or resource	Designation	Source or reference	Identifiers	Additional information
Cell line (Human)	HeLa	Imaging Facility, IFOM-IEO Campus, Milan, Italy		De Antoni et al., 2012. DOI: 10.1083/jcb.201205119
Cell line (Human)	mCherry-H2B HeLa	Imaging Facility, IFOM-IEO Campus, Milan, Italy		De Antoni et al., 2012. DOI: 10.1083/jcb.201205119
Cell line (Human)	RPE-Tir1	Kindly provided by the laboratory of Prof. Don Cleveland		Holland et al., 2012.DOI: 10.1073/pnas.1216880109
Cell line (Human)	U2OS	Kindly provided by the laboratory of Dr. Alex Bird		
Cell line (Human)	HEK293	Kindly provided by the laboratory of Dr. Alex Bird		
Cell line (Canine)	MDCK	Kindly provided by the laboratory of Dr. Yao-Wen Wu		Voss et al., 2016. DOI: 10.1073/pnas.1613999113
Cell line (Human)	A2780	Kindly provided by the laboratory of Dr. Philip Selenko		Theillet et al., 2016. DOI: 10.1038/nature16531
Cell line (Human)	RCSN3	Kindly provided by the laboratory of Dr. Philip Selenko		Theillet et al., 2016. DOI: 10.1038/nature16531
Cell line (Human)	B65	Kindly provided by the laboratory of Dr. Philip Selenko		Theillet et al., 2016. DOI: 10.1038/nature16531
Cell line (Human)	SKNSH	Kindly provided by the laboratory of Dr. Philip Selenko		Theillet et al., 2016. DOI: 10.1038/nature16531
Cell line (Human)	SHSY5Y	Kindly provided by the laboratory of Dr. Philip Selenko		Theillet et al., 2016. DOI: 10.1038/nature16531
Recombinant DNA reagent	pBIG1 with NDC80C-9A (NDC80C9A-GFP and SPC25-HIS)	This paper		See Materials and methods
Recombinant DNA reagent	pBIG1 with CFP-MIS12C (CFP-PMF1 and Dsn1-HIS)	This paper		See Materials and methods
Recombinant DNA reagent	pFl- BUBR11-571(∆432–484) (HIS-BUBR1mTurquoise2)	This paper		See Materials and methods
Sequence-based reagent	siRNA for Dsn1	Sigma-Aldrich		GUCUAUCAGUGUCGAUUUA
Sequence-based reagent	siRNA for Nsl1	Sigma-Aldrich		CAUGAGCUCUUUCUGUUUA
Sequence-based reagent	siRNA for Mis12	Sigma-Aldrich		GACGUUGACUUUCUUUGAU
Peptide, recombinant protein	MAD2-TAMRA	Musacchio laboratory		Faesen et al., 2017. DOI: 10.1038/nature21384
Peptide, recombinant protein	CFP-BUBR11-571	Musacchio laboratory		Faesen et al., 2017. DOI: 10.1038/nature21384
Peptide, recombinant protein	NDC80C-GFP	Musacchio laboratory		Weir et al., 2016. DOI: 10.1038/nature19333
Peptide, recombinant protein	SPINDLY-GFP/mCherry	Musacchio laboratory		Mosalaganti et al., 2017. DOI: 10.1083/jcb.201611060
Peptide, recombinant protein	EGFP-K-RAS D30C Tf3	Kindly provided by the Wu's laboratory		Voss et al., 2016. DOI: 10.1073/pnas.1613999113
Peptide, recombinant protein	α-Synuclein	Kindly provided by the Selenko's Laboratory		Theillet et al., 2016. DOI: 10.1038/nature16531
Commercial assay or kit	NEON Transfection System	Thermo Fisher	MPK5000S	
Commercial assay or kit	NEON Transfection 100 µl Kit	Thermo Fisher	MPK10025	
Commercial assay or kit	Nucleofector Device	Lonza	AAB-1001	
Chemical compound, drug	SiR-DNA kit	SphiroChrome	SC007	
Software, algorithm	Imaris 9	Bitplane		

### Electroporation of living cells

Electroporation (EP) was performed using either the Neon Transfection System Kit (Thermo Fisher) or the Amaxa Nucleofector I system (Lonza). For EPs with Neon Transfection System, cells were harvested by trypsinisation, washed with PBS, and resuspended in the electroporation Buffer R (Thermo Fisher) to a final volume of 90 µl. Between 2 and 3 million cells per electroporation were used in this study. Protein samples were diluted 1:1 in buffer R and then a volume of 30 µl of this mix was added to the cell suspension (the EP slurry). Volumes and protein-buffer ratios may be adjusted according to the purpose of the experiment and depending on protein solubility. Final protein concentrations in the respective EP slurry varied between 5 and 200 µM (in a final volume of 110 μL). The EP slurry was tipically loaded into a 100 µl Neon Pipette Tip (Thermo Fisher) and electroporated with two consecutive pulses at 1000V and for durations of 35 msec (with the exception of the experiments in U2OS, RPE and HEK293 cells, which were electroporated with 2 × 25 msec pulses at 800V). Following EP, the slurry was added to 50 ml of pre-warmed PBS, pelleted by a 0.5xg centrifugation and trypsinised for 5 to 7 min to remove non-internalized extracellular protein. Following one further wash in PBS and centrifugation, the cell pellet was re-suspended in complete imaging medium (without antibiotics) and transferred to a cell-imaging plate (Ibidi). Cells were returned to the incubator and allowed to recover for a minimum of 4 hr. After recovery, cells were either analyzed by fluorescence microscopy imaging or processed to generate protein extracts to be used for either immunoprecipitation analysis or western blotting. EP with the Amaxa Nucleofecto I system was performed according to the reported protocol (Theillet et al., 2016).

EP conditions described above gave satisfactory results for all the cell types and proteins tested. Only a small fraction of the tested recombinant proteins proved unsuitable for EP delivery. This was due to formation of major intracellular aggregates or because the protein samples failed to localize correctly within the cell. However, for maximal performance, we advise to optimize initially the EP pulse’s parameters (voltage strength, duration, and number of repetitions), the EP buffer composition, and the concentration of recombinant protein in the EP slurry (Figure 1—figure supplement 2C–H and Figure 2—figure supplement 1). Cell-specific and protein-specific optimization of such parameter should be always carried out in order to achieve the best trade-off between cell viability and efficient protein delivery. Protein specific optimization of EP voltage is particularly important for those cell lines, such as RPE1, U2OS and HEK293, whose viability is more sensitive to high EP voltages. The following are some of the important factors to consider during optimization of protein delivery: 1) In our work, we invariably used protein samples of great purity and homogeneity (high monodispersity and solubility). Although we did not systematically analyze the relationship between protein homogeneity and efficiency of EP, we suspect that protein sample quality is an important factor. 2) Centrifugation of protein sample prior to EP is strongly recommended. 3) During the recovery phase, we recommend avoiding the use of selection antibiotics in the media as they increase cell mortality. 4) Once resuspended in EP buffer, cells should be electroporated and washed as fast as possible to reduce cytotoxicity. 5) If required, including additional trypsinization and washing steps following EP may improve the removal of persistent extracellular protein aggregates generated during EP.

### Production of recombinant proteins

All recombinant proteins used in this study were of human origin. For expression and purification of recombinant proteins, synthetic codon-optimized DNA encoding human MAD2, BUBR1, SPINDLY, NDC80C and MIS12C subunits were used. All proteins used in this study were full length, except mTurquoise2-BUBR1^1-571^ (which we refer to as CFP-BUBR1^1-571^) and mTurquoise2-BUBR1^1-571(∆432–484)^ (which we refer to as CFP-BUBR1^1-571-∆Helix^). Fluorescent MAD2^TAMRA^, CFP-BUBR1^1-571^, mCherry/GFP-Spindly constructs, NDC80C^WT^-GFP complex and EGFP-K-RAS D30C Tf3 were expressed, purified, and labeled as previously described (Faesen et al., 2017; Mosalaganti et al., 2017; Weir et al., 2016; Voss et al., 2016). Recombinant NDC80C^9A^-GFP complex was generated by fusing a C-terminal GFP and a His6-tag to SPC25. Construct for insect cell expression exploited the MultiBac baculovirus expression system (Bieniossek et al., 2012). A Bacmid was then produced from EMBacY cells and subsequently used to transfect Sf9 cells and produce baculovirus. Baculovirus was amplified through three rounds of amplification and used to infect Tnao38 cells. Cells infected with virus were cultured for 72 hr before harvesting. Cell pellets were resuspended in buffer A (50 mM HEPES pH 8, 200 mM NaCl, 20 mM Imidazole, 5% glycerol, 2 mM TCEP) supplemented with protease-inhibitor mix (Serva) and 0.2% Triton, lysed by sonication and cleared by centrifugation. NDC80C^9A^-GFP was then eluted in buffer B (50 mM HEPES pH 8, 200 mM NaCl, 300 mM Imidazole, 5% glycerol, 2 mM TCEP) and the eluate was diluted six times in volume using ion exchange buffer C (50 mM HEPES pH 8, 25 mM NaCl, 5% glycerol, 2 mM TCEP, 1 mM EDTA) and applied to a 6 mL Resource Q anion-exchange column pre-equilibrated in the same buffer. Elution of bound protein was achieved by a linear gradient (25–400 mM NaCl in 25 column volumes). Relevant fractions were concentrated in 10 kDa molecular mass cut-off Amicon concentrators and applied to a Superose 6 16/70 column equilibrated in size-exclusion chromatography buffer D (50 mM HEPES pH 8, 250 mM NaCl, 5% glycerol, 2 mM TCEP). Peak fractions containing the NDC80C^9A^-GFP complex were collected and further concentrated in a 10 kDa cut-off Amicon concentrator before being flash frozen in liquid N2 and stored at −80°C. Recombinant CFP-MIS12 complex was generated by fusing N-terminal CFP to PMF1. Baculoviral transfer vectors encoding CFP-Mis12C expression were generated using biGBac platform (Weissmann et al., 2016). Baculoviruses were generated in Sf9 cells, and expression of CFP-tagged MIS12C was carried out in Tnao38 cells for 72 h-96h, at 27°C. CFP-Mis12C was purified by a three-step protocol, as described previously for the non-fluorescent version (Petrovic et al., 2016): i) affinity purification of filtered supernatant with 5 ml HisTrap FF column (GE Healthcare) and step elution with 300 mM imidazole; ii) anion-exchange of the dialyzed eluate with 6 ml Resource Q column, elution with linear NaCl gradient; and iii) final polishing step via size-exclusion on a Superdex 200 10/300 column (GE Healthcare) equilibrated in 20 mM Tris-HCl (pH 8.0), 0.15 M NaCl, and 1 mM TCEP. Relevant fractions were pooled, concentrated, flash-frozen in liquid nitrogen and stored at −80°C. Recombinant CFP-BUBR1^1-571-∆Helix^ was expressed in Tnao38 cells that were then lysed in buffer A (25 mM HEPES (pH 7.5), 300 mM NaCl, 10% glycerol, 2 mM TCEP, 1 mM PMSF). Soluble lysates were passed over a 5 ml Ni-NTA column and, after washing with 20 column volumes buffer A, the proteins were eluted by adding 300 mM imidazole to buffer A. Proteins were subsequently gel-filtered on a Superdex S200 16/60 column equilibrated against buffer B (10 mM HEPES (pH 7.5), 150 mM NaCl, 5% glycerol, 2 mM TCEP). Fractions containing purified protein were concentrated, flash-frozen and stored at −80°C. Recombinant N-terminally acetylated human α-SYNUCLEIN (αSYN) was purified from *E. coli* as reported (Theillet et al., 2016). In vitro pre-farnesylation of mCherry-Spindly (30 µM) was achieved by incubation with recombinant Farnesyltransferase (10 μM) and farnesylpyrophosphate (90 µM) for 5–6 hr at 20°C, followed by gel filtration (S200) purification to remove the Farnesyltransferase.

### Cell culture, siRNA transfection, immunoprecipitation, immunoblotting, analysis of intracellular protein levels and viability assay

The following cell lines were cultured in DMEM (PAN Biotech) supplemented with 10% FBS (Clontech), penicillin, streptomycin (GIBCO) and 2 mM L-glutamine (PAN Biotech): HeLa, mCherry-H2B HeLa, U2OS, MDCK, HEK293, and RPE-Tir1. The following cell lines were grown in the following media (supplemented as above): Human A2780 and B65 in RPMI 1640, SK-N-SH and RCSN3 DMEM-Ham’s F-12 and SH-SY5Y in DMEM. Cells were grown at 37°C in the presence of 5% CO_2_. All experiments requiring live imaging were performed in complemented CO_2_-independent medium (GIBCO) at 37°C. Cell lines were not further authenticated. Cells used in this study are regularly checked for mycoplasma contamination and test negative. Unless differently indicated, the microtubule-depolimerising drug nocodazole was used at 3.3 μM (Sigma). Endogenous farnseyltransferase inhibition was achieved at 10 μM of FTI-277 (Sigma). Cellular RAS activity was stimulated with 50 ng/ml EGF (Sigma). Were indicated, the DNA dye SiR-Hoechst-647 Dye (Spirochrome) at a concentration of 0.5 µM was added to the medium 1 hr before live imaging. Depletion of endogenous MIS12C was achieved by RNAiMax (Invitrogen) transfection of 3 combined siRNA duplexes used at 10 nM each for 48 hr (RNA oligos sequence for Dsn1 is GUCUAUCAGUGUCGAUUUA; for Nsl1 is CAUGAGCUCUUUCUGUUUA; Sigma-Aldrich) (RNA oligos sequence for MIS12 is for GACGUUGACUUUCUUUGAU; GE Healthcare Dharmacon). To generate mitotic populations for immunoprecipitation experiments after EP, cells were treated with nocodazole for 16 hr. Mitotic cells were then harvested by shake off and resuspended in lysis buffer [150 mM KCl, 75 mM Hepes, pH 7.5, 1.5 mM EGTA, 1.5 mM MgCl2, 10% glycerol, and 0.075 % NP-40 supplemented with protease inhibitor cocktail (Serva) and PhosSTOP phosphatase inhibitors (Roche)]. A total of 4 mg of protein extract per sample was then incubated with GFP-Traps beads (ChromoTek; 3 μl/mg of extract) for 3 hr at 4°C. Immunoprecipitates were washed with lysis buffer and resuspended in sample buffer, boiled and analyzed by SDS- PAGE and Western blotting using 4–12% gradient gels (NuPAGE). The following antibodies were used for the western blot analysis in this study: anti-Bub1 (rabbit polyclonal; Abcam9000; 1:5000), anti-Hec1 (human Ndc80; mouse clone 9G3.23; Gene- Tex, Inc; 1:250), anti-Mis12 (in house made mouse monoclonal antibody; clone QA21; 1:1000), anti-GFP (in house made rabbit polyclonal antibody; 1:1,000–4,000) anti-Vinculin (mouse monoclonal; clone hVIN-1; Sigma-Aldrich; 1:10000), anti PMF1/NNF1 (in house made mouse affinity purified monoclonal; clone RH25-1-54, 1:1000) and anti-Tubulin (mouse monoclonal, Sigma-Aldrich; 1:10000). Quantification of protein levels from western blots was performed with the following formula: [(LPoIsiRNA-PoIBgr)/(LVincsiRNA-VincBgr)]/[(LPoICtrl-PoIBgr)/(LVincCtrl-VincBgr)]. LPoIsiRNA = levels of the protein of interest for the siRNA lane; PoIBgr = background signal for the protein of interest; LVincsiRNA = levels of Vinculin for the siRNA lane; VincBgr = background signal for Vinculin; LPoICtrl = levels of the protein of interest for the Control lane; PoIBgr = background signal for the protein of interest; LVincCtrl = levels of Vinculin for the Control lane; VincBgr = background signal for Vinculin. Fluorimetric analysis was performed using Greiner flat-bottom plates and a Clariostar microplate reader (monochromator excitation at 587 ± 10 nm, emission 610 ± 10 nM). Fluorescence intensity from protein extracts derived from a known number of electroporated cells were measured and plotted against a calibration curve generated with defined concentrations of recombinant mCherry. Bar graphs show average intracellular concentrations and SD for two independent experiments in which every concentration was analysed in duplicate. For viability assays, the percentage of viable cells was measured by Trypan Blue staining followed by automatic counting of viable cells using the Countess automated cell counter (Thermo Fisher). Each sample was counted twice and values showed in figures represents the average of these counts. For the viability assay comparing different EP buffers, cells and protein were resuspended in the following buffers: 1) Buffer R, provided in the commercial EP kit from Thermo Fisher; 2) PBS, 5% Glycerol, 1 mM MgCl2, 2 mM TCEP; 3) 50 mM Tris pH 7.5, 5% Glycerol, 125 mM NaCl, 1 mM MgCl2, 2 mM TCEP; 4) 25 mM HEPES pH 7.5, 5% Glycerol, 125 mM NaCl, 1 mM MgCl2, 2 mM TCEP.

### α-SYNUCLEIN detection and flow cytometry

For immunofluorescence imaging of delivered αSYN in fixed samples (electroporation concentration 400 µM), cells were recovered for 5 hr in the incubator on poly-L-lysine-coated 25 mm cover slips. Cells were quickly washed 3 × with complete medium and treated briefly with diluted trypsin/EDTA (0.01 %/ 0.004%, 40 s, room temperature) to remove non-internalized αSYN, then fixed in PBS containing 4% (w/v) PFA for 15 min and permeabilized with 0.1% (v/v) Triton-X in PBS for 3 min. After washing 3 × 10 min with PBS, samples were blocked with 0.13% (v/v) cold fish skin gelatin (Sigma) in PBS for 1 hr. Cells were incubated for 2 hr with anti-αSYN ab52168 (Abcam, 1:100 dilution) in blocking buffer. After washing 3 × 10 min with PBS, specimens were incubated with anti-mouse IgG Atto647, (Sigma, 1:1000 dilution) and fluorescein isothiocyanate (FITC)-labeled phalloidin (Millipore, 2 mg/mL) for 1 hr in blocking buffer. Slides were washed 3 × 10 min with PBS and nuclei stained with 1 µg/mL 4',6-diamidino-2-phenylindole (DAPI, Invitrogen) in PBS for 15 min. After washing once in PBS, samples were mounted with Immu-Mount (Thermo Scientific). For αSYN detection by western blotting, cell lysates were separated on commercial 4–18% gradient SDS-PAGE (BioRad), transferred onto PVDF membranes and fixed with 4% (w/v) paraformaldehyde (PFA) in PBS for 1 hr. Membranes were washed 2 × 5 min with PBS and 2 × 5 min with TBS (25 mM Tris, 136.9 mM NaCl, 2.7 mM KCl, pH 7.4). After blocking for 1 hr in 5% (w/v) milk in TBST (0.1% (v/v) Tween-20 in TBS), membranes were probed with anti-αSYN sc69977 (Santa Cruz, 1:100 dilution) and anti-Actin IgM (Merck Millipore, JLA20, 1:5000 dilution). Secondary antibodies were HRP-conjugated anti-mouse or anti-rabbit (Sigma, 1:10,000 dilutions). Membranes were developed using SuperSignal West Pico or Femto chemiluminescent substrates (Thermo Scientific). Luminescence signals were detected on a BioRad Molecular Imager and quantified with ImageLab (BioRad). Flow-cytometry and quantification (fluorescence intensity median analysis) of αSYN containing cells were performed with lysine-to-Alex488 fluorophore-coupled recombinant protein as described (Theillet et al., 2016). In brief, N-hydroxysuccinimide (NHS) ester-activated Atto488 fluorescent dye (Sigma Aldrich) was coupled to αSYN lysine side chain-amines in bicarbonate buffer at pH 8.3 according to the manufacturer’s instructions. Covalently modified αSYN was separated from non-reacted dye on a Sephadex G-25 column (Amersham, GE) and concentrated with 6 kDa MW cut-off spin columns (Millipore). The final concentration of the fluorescently labeled protein was measured by UV-VIS spectrophotometry at 280 nm with E: 5690 M^−1^cm^−1^. A correction factor of 0.1 was subtracted to compensate for the intrinsic absorbance of the fluorophore. Cells containing Atto488-tagged protein (530 nm) were detected by flow cytometry on a FACSCalibur (BD Biosciences,>10,000 recorded events for each sample). Median αSYN fluorescence from all events was determined with FlowJo 8.8.6 for each sample.

### Microscopy, immunofluorescence detection, live-cell imaging, FRAP analysis, FRET-FLIM imaging and chemical inhibition

For this study, the following microscopes were used. 1) A customized 3i Marianas system (Intelligent Imaging Innovations) equipped with a Axio Observer Z1 microscope chassis (Zeiss), a CSU-X1 confocal scanner unit (Yokogawa Electric Corporation), Plan-Apochromat 63×/1.4 NA objectives (Zeiss), an Orca Flash 4.0 sCMOS Camera (Hamamatsu), and a FRAP Vector High Speed Point Scanner. Images were acquired as *Z* sections (using Slidebook Software six from Intelligent Imaging Innovations) and converted into maximal-intensity-projection TIFF files for illustrative purposes. 2) A Deltavision Elite System (GE Healthcare) equipped with an IX-71 inverted microscope (Olympus), a UPlanFLN 40×/1.3 NA objective (Olympus) and a pco.edge sCMOS camera (PCO-TECH Inc). Images were acquired as *Z* sections (using the softWoRx software from Deltavision) and converted into maximal-intensity-projection TIFF files for illustrative purposes. 3) An Olympus FV1000 FlouView laser scanning confocal microscope counted in a single-photon counting avalanche photodiode (PDM Series, MPD; PicoQuant) and timed by using a time-correlated single-photon counting module (PicoHarp 300; PicoQuant) after being spectrally filtered using a narrow-band emission filter (HQ 525/15; Chroma). The microscope was also equipped with an Eppendorf Transjector 5246 and an Eppendorf Micromanipulator 5171. 4) For αSYN experiments, confocal images were taken at 40 × magnifications and using excitation wavelengths of 405, 488, and 633 nm on a Zeiss LSM 510 META laser-scanning microscope. For immunofluorescence analysis, cells (HeLa) were grown on coverslips pre-coated with 15 µg/ml poly-d-lysine (Millipore) and then fixed with 4% paraformaldehyde in either PBS or PHEM, followed by permeabilization with either PBS or PHEM containing Tween 0.3%. The following antibodies were used for immunostaining: anti-CREST/anti-centromere antibodies (human, Antibodies Inc; 1:100) anti-Tubulin (mouse monoclonal, Sigma; 1:5000). DNA was stained with 0.5 µg/ml DAPI (Serva) and coverslips mounted with Mowiol mounting medium (Calbiochem). Quantification of kinetochore signals was performed on unmodified 16-bit z-series images using Imaris nine software (Bitplane). After background subtraction, all signals were normalized to CREST signal. Measurements were plotted with GraphPad Prism 6.0 (GraphPad Software).

The individual electroporation of HeLa cell with either the CFP-BUBR1^1-571^ constructs or MAD2^TAMRA^ was performed using an EP concentration of respectively 10 µM and 5 µM. After electroporation cells were allowed to recover overnight and then treated with nocodazole for imaging on the Mariana system. To determine CFP kinetochore levels for Figure 1D, between 263 and 372 KTs per condition were scored. Simultaneous electroporation of 10 µM CFP-BUBR1^1-571^ and 5 µM MAD2^TAMRA^ was performed on HeLa cells and imaged live on a Deltavision system. To measure mitotic duration in cells depleted for endogenous MIS12C and electroporated with recombinant CFP-MIS12C, at either 8, 5 or 2.5 µM, H2B-mCherry HeLa cells were transfected with siRNA-MIS12C oligos, twice in 24 hr, and then electroporated 6 hr after the second transfection. Following EP, cells recovered overnight before being processed for either western blotting analysis or imaged lived on the Deltavision system. Mitotic duration, with or without 330 nM nocodazole, was assessed by DNA morphology. Cells entering anaphase with their DNA not completely aligned in the metaphase plate were scored as erroneous segregations. Between 53 and 149 cells per condition were scored. Live-cell time-lapse imaging of HeLa cells electroporated with NDC80C^WT^-GFP at 5 µM was performed on an asynchronously growing population. Cells were then left to recover overnight before being treated with SiR-Hoechst-647 Dye for one hour before being imaged live on a Deltavision system. To determine mitotic duration, between 60 and 70 cells per condition were scored. For the error correction assay, following electroporation HeLa cells were synchronized in G2 with 9 µM RO-3306 (Millipore) for 16 hr and then released in the presence of 5 µM STLC (Sigma-Aldrich) for 3 hr. Following STLC wash-out, cells were grown for 150 min in media containing 5 µM MG132 (Calbiochem), then fixed, prepared for immunofluorescence analysis and then scored for the presence of uncongressed chromosomes on the Mariana microscope. Cells with one or more chromosomes not perfectly aligned at the metaphase plate were scored as uncongressed. Results are representing the average ± SD of two replicated experiments. In total, between 140 and 191 cells were scored for each condition. Electroporation of NDC80C^WT^-GFP into RPE1, U2OS or HEK293 cells, was performed on asynchronously growing cultures. Following electroporation cells were left recover overnight and then incubated in nocodazole and SiR-Hoechst-647 Dye for 4 hr, and then imaged live on the Mariana microscope. For Spindly experiments, asynchronously growing HeLa cells were treated with farnesyltransferase inhibitor FTI for 24 hr before being electroporated with fluorescent Spindly at 10 µM. Following EP, cells were left to recover for 24 hr in FTI-277, of which the last 6 hr were in the presence of nocodazole. Immunofluorescence analysis was performed on the Mariana system. To determine kinetochore levels for fluorescent SPINDLY, between 60 and 220 KTs per condition were scored. For EGFP-KRAS D30C-TF3 experiments, microinjections and electroporations for FLIM-FRET imaging were performed on MDCK cells that were serum-starved overnight in MEM Eagle medium (Sigma) supplemented with 0.5% FBS. Following starvation, cells were either microinjected or electroporated with EGFP-KRAS D30C-TF3 at a concentration of 6–10 mg/mL and then left recover for 6 hr before live-cell imaging by FLIM-FRET microscopy. Microinjections and FLIM-FRET imaging were performed on an Olympus FV1000 as previously described (Voss et al., 2016). FLIM-FRET analysis of EGFP-KRAS D30C-TF3 lifetime was performed on 12 electroporated cells.

## Data Availability

All data generated or analysed during this study are included in the manuscript and supporting files.

## Additional files

Transparent reporting form
