# Supplementary material for: Electroporated recombinant proteins as tools for in vivo functional complementation, imaging and chemical biology
Source: eLife. 2019 Jul 16;8:e48287. doi: 10.7554/eLife.48287 (PMC6656429; doi:10.7554/eLife.48287)
Supplement: Peer review history

# Decision letter

**DOI:** 10.7554/eLife.48287.023

**Affiliations:** Virginia TechUnited States; University of KonstanzGermany

In the interests of transparency, eLife includes the editorial decision letter and accompanying author responses. A lightly edited version of the letter sent to the authors after peer review is shown, indicating the most substantive concerns; minor comments are not usually included.

Thank you for submitting your article "Electroporated recombinant proteins as tools for in vivo functional complementation, imaging, and chemical biology" for consideration by *eLife*. Your article has been reviewed by three peer reviewers, one of whom is a member of our Board of Reviewing Editors, and the evaluation has been overseen by Anna Akhmanova as the Senior Editor. The following individuals involved in review of your submission have agreed to reveal their identity: Yoshio Kato, Thomas Mayer, Aaron Straight.

The reviewers have discussed the reviews with one another and the Reviewing Editor has drafted this decision to help you prepare a revised submission.

Summary:

Alex, Maffini, Musacchio et al. report on a systematic assessment of electroporation as a method to deliver recombinant proteins or protein complexes into mammalian cells. Electroporation as a technique for protein delivery is not novel, but the authors demonstrate in this manuscript that the technique is less disruptive and provides more homogenous outcomes across a cell population than previously thought.

The reviewers found that the work is very well performed and described. Important controls are included, e.g. different cell lines, titration of amount of protein used for electroporation etc. Importantly, thorough functional studies confirm that the electroporated proteins are functional and can complement the loss of endogenous proteins. The authors demonstrate that entire protein complexes can be electroporated and remain functional, and they use in vitro farnesylation to demonstrate that electroporation can introduce modified recombinant proteins into living cells. The authors also show that the efficiency of delivery is high enough to conduct bulk assays like immunoblotting or immunoprecipitation.

In summary, the authors convincingly demonstrate that electroporation has unrecognized potential as a technique, as it can deliver chemically modified proteins that cannot be easily produced in cells and allows for sophisticated cell biological or biochemical follow-up experiments. The paper is important as it will make the technique more widely known and attractive for other researchers to try.

Essential revisions:

1) Since the paper is meant to make the technique accessible to others, some additional information will be useful:

- Please provide more information on how settings in the electroporation procedure or other relevant parameters affect the efficiency of delivery, viability of cells etc.

- In addition to the phase contrast microscopy (Figure 1—figure supplement 1F), a viable/dead stain to quantify the extent of damage or toxicity in the different cell lines would be useful.

- Please add data for RPE1 and HEK293, which seem to be more difficult to electroporate (mentioned in the Materials and methods section).

- While it is great to have the successes of the technique highlighted, the discussion of shortcomings is equally valuable. Please move the discussion of potential shortcomings to the main section of the manuscript (e.g. Discussion), where it will be easier to find than in the Materials and methods section.

- In the Materials and methods section, it would be useful to mention which number or concentration of cells was used in the respective volumes (subsection “Electroporation of living cells”).

- It may not be possible for the authors to do anything about this (since this seems proprietary information), but it would be useful to know the composition of "buffer R". Otherwise, it is hard to know how conditions can be adjusted if a protein of interest happens to be incompatible. Have the authors tried buffers of known composition and can comment?

2) In Figure 2A, the levels of ectopic MIS12 are much higher than the ones of endogenous Mis12. Are such high levels of ectopic protein required to complement the loss of endogenous Mis12? Please comment.

# Reply: Author response

**DOI:** 10.7554/eLife.48287.024

Essential revisions:

1) Since the paper is meant to make the technique accessible to others, some additional information will be useful:

- Please provide more information on how settings in the electroporation procedure or other relevant parameters affect the efficiency of delivery, viability of cells etc.

We agree with the reviewers that further information on the electroporation settings would make the method more accessible to others. We therefore added a new figure (Figure 1—figure supplement 2) where we report the effects on cell viability and efficiency of protein uptake from changing several parameters, such as the EP pulse settings (voltage strength, duration, and number of repetitions) or the EP buffer. Specifically, we measured 1) how different voltages of the EP pulse affect the viability and protein uptake in different cell lines; 2) the effect of the number of electrical pulses on the viability and protein uptake of different cell lines at different time points; and 3) how electroporation buffers of known composition (such as Tris, or Hepes or PBS based buffers) affect viability and uptake in comparison with the proprietary buffer R (which achieved the best trade-off between viability and efficiency of delivery.

Following the reviewers’ suggestion, we now discuss “shortcomings”, in the main text of the manuscript. An important limitation is that some instrumental settings are instrument-specific and, for the systems used in this study (the NEON and the Nucleofector I), represent undisclosed proprietary information to which we have no access. The electroporation system Nucleofector I (which was used only for the EP of α-SYNUCLEIN) provides only predefined EP “programs” for which no information on voltage, duration of the pulses used and composition of the EP buffer are available. The NEON system allows for the control of the EP settings (voltage strength and length of the pulse) but employs proprietary electroporation buffers for which the composition is not disclosed. Electroporation systems that provide more control on the electroporation settings and that do not require special EP buffers, such as the NEPA21, exists commercially but were not available to us.

- In addition to the phase contrast microscopy (Figure 1—figure supplement 1F), a viable/dead stain to quantify the extent of damage or toxicity in the different cell lines would be useful.

We thank the reviewers for this suggestion. As mentioned above, we now included viability assays performed with Trypan blue staining followed by automatic counting of viable cells using the Countess automated cell counter.

- Please add data for RPE1 and HEK293, which seem to be more difficult to electroporate (mentioned in the Materials and methods section).

New viability measurements shown in Figure 1—figure supplement 2 indicate that the viability of RPE, HEK293 and U2OS cells is affected when electroporation is performed at higher voltages.

- While it is great to have the successes of the technique highlighted, the discussion of shortcomings is equally valuable. Please move the discussion of potential shortcomings to the main section of the manuscript (e.g. Discussion), where it will be easier to find than in the Materials and methods section.

We thank the reviewer for this suggestion. We have re-positioned a description of the shortcoming of the method into the main body of the manuscript, in the section titled *Electroporation: an outline*, where we describe the general procedure and discuss the optimization of EP parameters.

- In the Materials and methods section, it would be useful to mention which number or concentration of cells was used in the respective volumes (subsection “Electroporation of living cells”).

We thank the reviewers for pointing out this important missing detail that has been now added to the subsection “EP of a protein complex with an exceptionally large hydrodynamic radius and its interaction with endogenous partners”.

- It may not be possible for the authors to do anything about this (since this seems proprietary information), but it would be useful to know the composition of "buffer R". Otherwise, it is hard to know how conditions can be adjusted if a protein of interest happens to be incompatible. Have the authors tried buffers of known composition and can comment?

As predicted by the reviewers, the composition of buffer R used for the NEON systems is undisclosed. We now describe more buffer options and information on their suitability in a test case (Figure 1—figure supplement 2). In terms of delivery efficiency, buffer R clearly performed better than the other tested buffers.

2) In Figure 2A, the levels of ectopic MIS12 are much higher than the ones of endogenous Mis12. Are such high levels of ectopic protein required to complement the loss of endogenous Mis12? Please comment.

We thank the reviewer for raising this important point. We have now included Figure 2—figure supplement 1 to report the results of an experiment in which cells depleted for endogenous MIS12 complex were electroporated with decreasing concentrations of recombinant CFP-MIS12 complex and its activity was assessed with our functional assays. We observed that full rescue of the MIS12 depletion phenotype requires intracellular levels of recombinant MIS12 that are slightly higher than the normal levels of the endogenous counterpart. This observation is now stated in the main text of the manuscript, together with a speculative explanation.
